# Mercury contamination impairs the hypothalamic-pituitary-adrenal axis of a neotropical caiman

**DOI:** 10.1038/s42003-026-11022-0

**Published:** 2026-10-28

**Authors:** Jérémy Lemaire, Rosanna Mangione, Jasmine L. Loveland, Leonida Fusani, Virginie Canoine

**Affiliations:** 1 Department of Behavioral and Cognitive Biology, University of Vienna, Vienna, Austria; 2 Department of Interdisciplinary Life Sciences, Messerli Research Institute, University of Veterinary Medicine, Vienna, Austria; 3 Konrad Lorenz Institute of Ethology, University of Veterinary Medicine, Vienna, Austria

**Keywords:** Behavioural ecology, Tropical ecology

## Abstract

The capacity to regulate corticosterone release following a stressful event is an essential component of the physiological flexibility required to maintain homeostasis and ensure adaptive responses to environmental change. We show that wild smooth-fronted caimans with elevated blood mercury (Hg) concentrations exhibited reduced peak corticosterone levels during standardized stress tests, indicating a dampened maximal stress response. Hg blood concentrations were negatively correlated with corticosterone responsiveness, suggesting impairment of hypothalamic–pituitary–adrenal axis function. These results demonstrate that environmental Hg contamination is associated with reduced stress responsiveness and physiological flexibility, which likely limits the ability of Hg-contaminated individuals to cope with novel environmental stressors. We found that a methylmercury-sensitive cysteine in the glucocorticoid receptor is highly conserved across vertebrates, suggesting a shared molecular mechanism for mercury effects on glucocorticoid signaling. Given the widespread Hg pollution in tropical ecosystems, these findings emphasize the role of Hg as an endocrine-disrupting chemical in crocodylians.

## Abstract (web summary)

Reduced physiological stress response is associated with elevated mercury from environmental contamination in wild caimans, supporting a conserved mechanism of endocrine disruption with implications for wildlife resilience.

## Introduction

Environmental pollution is one of the most pressing global changes threatening wildlife, ranking as the third most significant driver of biodiversity loss^1^. Mercury (Hg) contamination is a global anthropogenic threat to ecosystems due to its high toxicity on living organisms^2^. Since the pre-industrial era (1850–1900), environmental concentrations of Hg have risen drastically with modern fossil fuel-powered technologies estimated to have caused a 300–500% increase in atmospheric Hg levels above natural baseline^3^. Artisanal and Small-Scale Gold Mining (ASGM) activities are among the leading sources of Hg pollution because they consume over 2000 tons of Hg annually^4,5^. In tropical and subtropical regions, Hg is particularly concerning due to its natural prevalence and the intensification of ASGM activities, which account for more than 80% of Hg emissions in South America and Sub-Saharan Africa, representing 38% of global atmospheric Hg emissions^4^.

Historically, research on Hg effects has focused primarily on fishes, birds, and mammals, thereby advancing the understanding of its toxicity on wildlife, but leaving reptiles—and particularly crocodylians—understudied^6,7^. Notably, crocodylians often inhabit areas where ASGM is rampant, making them highly exposed to Hg contamination mainly through their diet^8,9^. Despite their resilience, crocodylians, which have withstood environmental changes for over 200 million years, are facing modern threats: over one-third of the 26 extant species are currently threatened by extinction according to the IUCN Red List^10^. The effects of endocrine-disrupting chemicals (EDCs) have long been identified as an ecological and toxicological concern in crocodylians^11^. Owing to their longevity and, therefore, susceptibility to bioaccumulate Hg over decades, crocodylians are crucial for understanding the chronic impacts of the contaminant on reptile endocrinology, a field that remains poorly understood^12^.

Mercury is recognized as an EDC that interferes with vertebrate reproductive systems throughout their lifetime, with embryonic and early postnatal exposures exerting the most potent effects^13^. Physiological alterations involve the hypothalamic-pituitary-adrenal (HPA) axis, which is important for stress management and behavioral flexibility, resulting in severe negative effects such as suppressed immune function, hormonal imbalance, reduced reproductive activity and consequently, reduced fitness^14^. Advancing knowledge on Hg’s effects on non-traditional model organisms and free-ranging species^15,16^—particularly those highly vulnerable to global changes, such as crocodylians—is critical for informing effective conservation strategies.

**Fig. 1:**
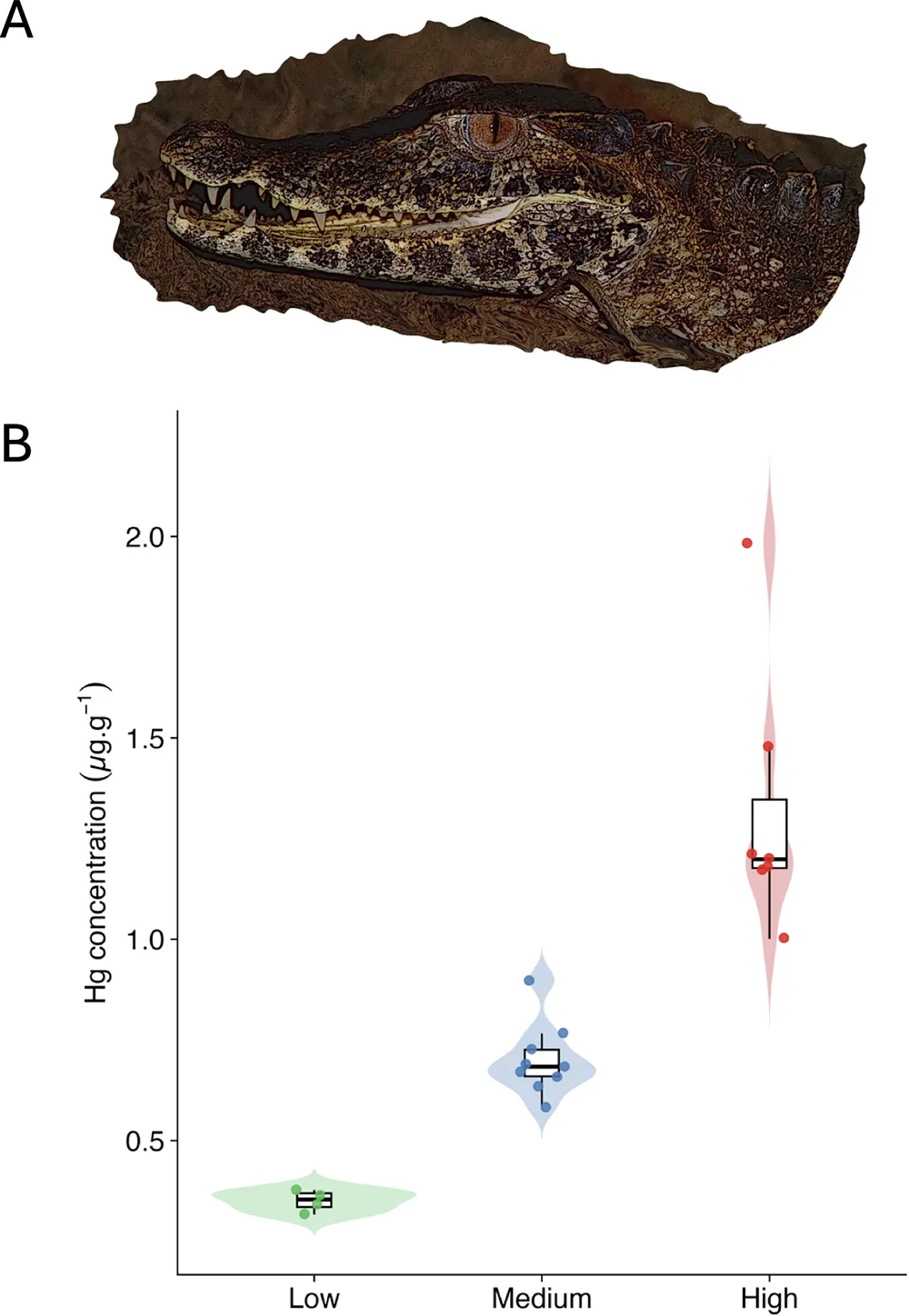
Mercury concentrations in red blood cells in smooth-fronted caimans used in this study. **A** Adult smooth-fronted caiman, *Paleosuchus trigonatus*. **B** Boxplot of mercury concentrations (in µg.g^−1^) in red blood cells of smooth-fronted caimans, *Paleosuchus trigonatus*, in French Guiana. High represents individuals with blood Hg > 1 µg.g^−1^ (*n* = 7); Medium, individuals with Hg between 0.5 and 1 µg.g^−1^ (*n* = 9), and Low individuals with Hg < 0.5. µg.g^−1^ (*n* = 4).

The life-history traits of crocodylians—oviparous, ectothermic, long-lived top predators—amplify chronic exposure to sublethal Hg concentrations throughout their lifespan^8^. As large-bodied predators of freshwater biomes, caimans play an essential role in the ecological dynamics of some of the planet’s most vulnerable ecosystems^17^. The smooth-fronted caiman, *Paleosuchus trigonatus* (Fig. 1A), a species inhabiting tropical rainforests heavily impacted by ASGM, is of particular concern due to lifelong dietary Hg exposure^9^. Understanding the severity of the effects of Hg contamination on its endocrine system is fundamental for assessing implications at both individual and population levels.

To investigate whether Hg contamination affects endocrine responsiveness, we measured the HPA response of wild smooth-fronted caimans in French Guiana in pristine rainforest and at ASGM-affected sites (*n* = 20). While a reduced corticosterone (CORT) response has been shown in Hg-exposed captive model organisms^18^, this is the first study to measure CORT responses in nature within a tropical ecosystem exposed to elevated Hg levels.

First, we quantified baseline levels of CORT, a key steroid hormone and major regulatory factor of the HPA axis essential for maintaining homeostasis^19^. CORT baseline concentrations may potentially reflect long-term exposure to stressors and are a good bioindicator of chronic stress^20,21^. Then, we quantified the acute response to stressors by using a classical serial sampling protocol, where capture and handling served as standardized stressors. We expected the stress response to be altered in individuals that had high levels of Hg, compared to those with lowest levels.

**Fig. 2:**
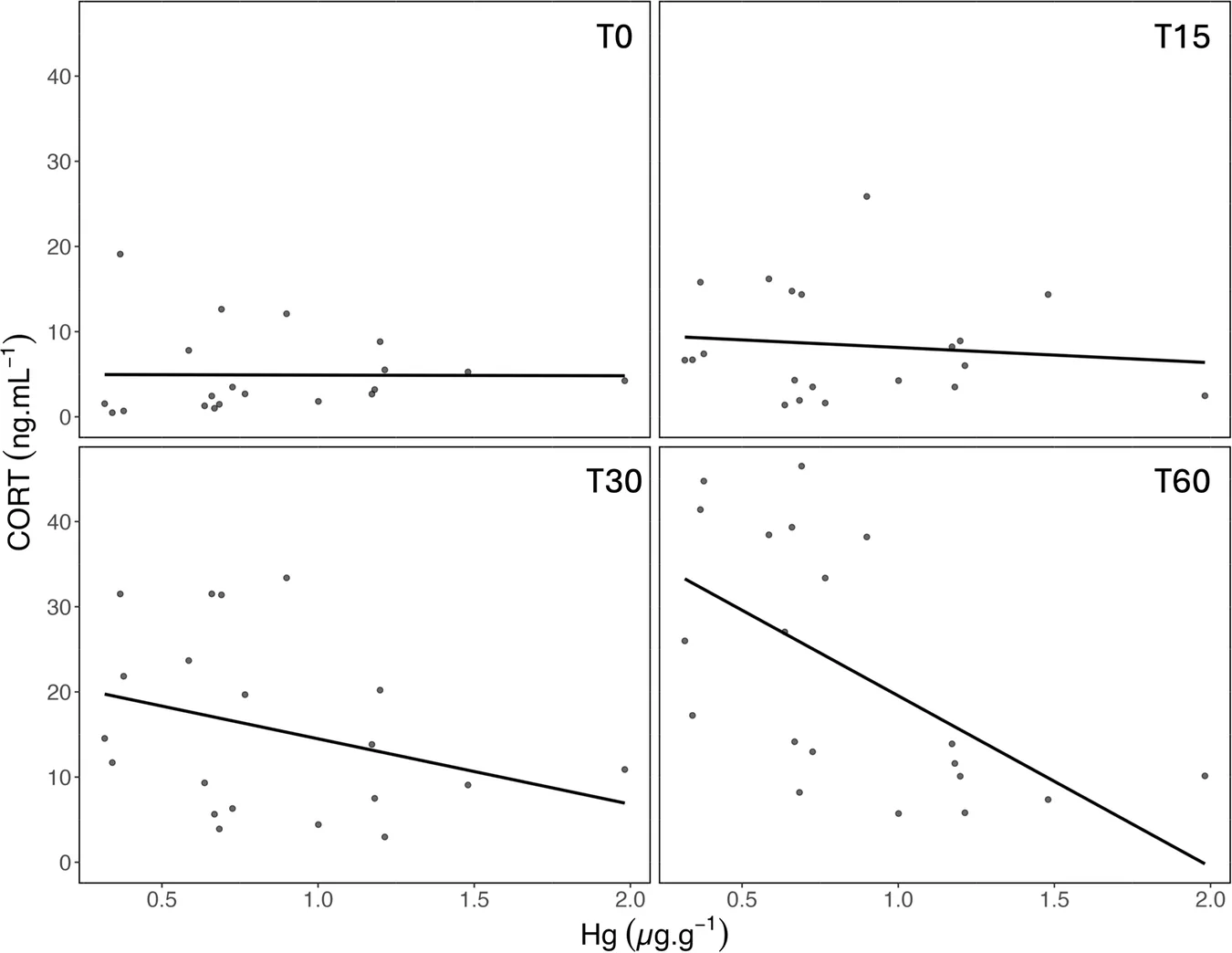
Effect of mercury concentration on corticosterone levels at different time points after capture. Relationship between Hg concentrations (µg.g^−1^) in red blood cells and plasma CORT levels (ng.mL^−1^) of smooth-fronted caimans, *Paleosuchus trigonatus*, in French Guiana at four time points of the serial sampling (*n* = 20). Dots represent raw data and lines represent predicted CORT levels from the linear mixed-effects model (CORT ~ Time * Hg + (1 | ID)). Mercury was significantly associated with decreased CORT levels at T60 (β = −20.009, SE = 4.181, t = −4.786, *p* < 0.001) but not at T0, T15 and T30 (*p* > 0.05).

**Fig. 3:**
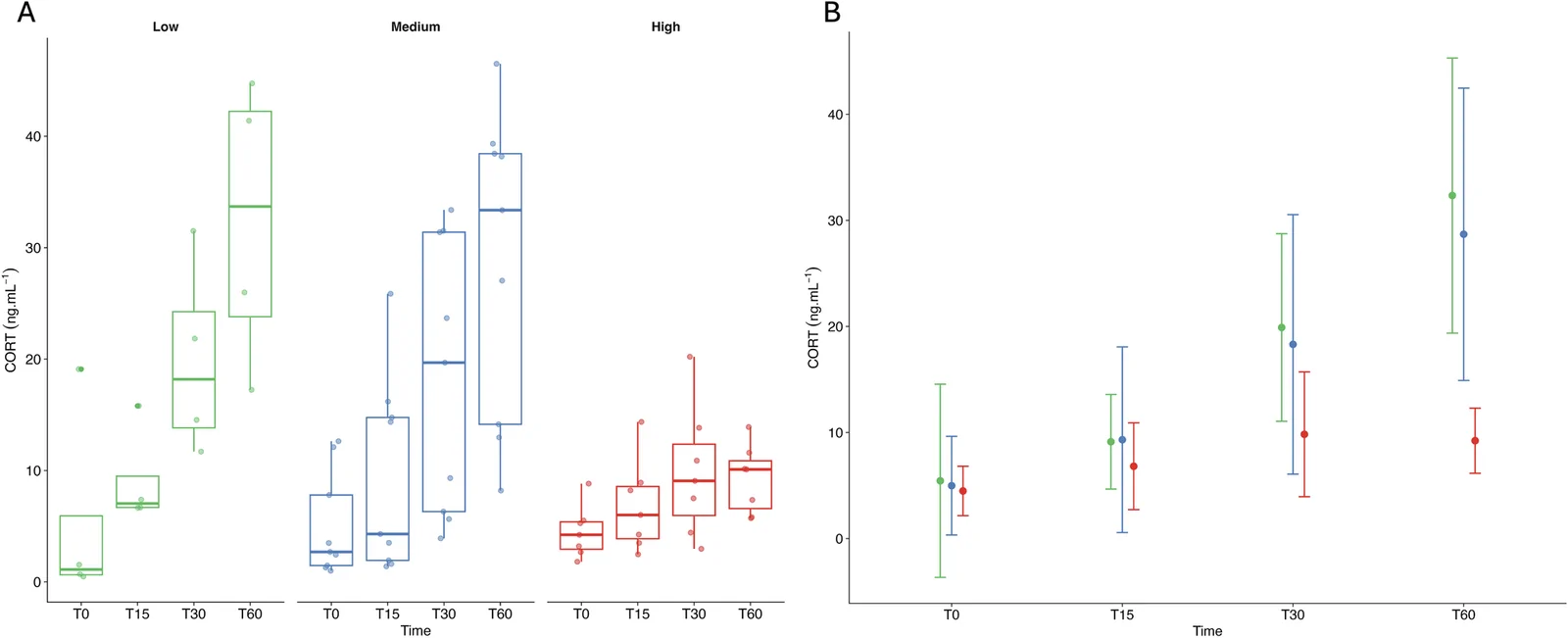
Serial sampling corticosterone levels in relation to mercury concentrations. **A** Boxplot of corticosterone concentrations (in ng.mL^−1^) in plasma of smooth-fronted caimans, *Paleosuchus trigonatus*, in French Guiana during a stress response test. Low are individuals with Hg < 0.5. µg.g^−1^ (*n* = 4); Medium, individuals with Hg between 0.5 and 1 µg.g^−1^ (*n* = 9); High represents individuals with blood Hg > 1 µg.g^−1^ (*n* = 7). Time is expressed in minutes. Significant difference for the CORT levels were found at 60 min after capture (T60) between the groups High and Low, and High and Medium (*p* < 0.05). **B** Relationship between Hg concentrations (µg.g^−1^) in red blood cells and amplitude of plasma corticosterone change from T0 to T60 (∆CORT, in ng.mL^−1^) (linear regression: R^2^ = 0.398, *p* = 0.003, *n* = 20).

We found that baseline CORT levels were similar across populations with various levels of Hg contamination (Figs. 1B, 2, 3A). However, the maximal stress response reached lower peak levels in Hg-contaminated individuals (Figs. 2, 3A). Further, the responsiveness towards a new stressor, such as capture by a predator (here simulated by the researcher), showed a strong negative correlation with Hg concentrations (Fig. 3B). These results strongly indicate that environmental Hg contamination affects the endocrine system and particularly the HPA axis in caimans by downregulating the stress response, with a reduced physiological plasticity to adequately respond to new stressors. These results suggest that increased Hg concentrations as those resulting from ASGM activities can profoundly impact caiman well-being and survival.

## Results

The linear mixed-effects model revealed no significant main effect of Hg concentration on CORT levels (β = 0.084, SE = 4.808, t = −0.017, *p* = 0.986). There was no significant main effect of time, with CORT levels at 15 min (β = 4.939, SE = 3.943, t = 1.252, *p* = 0.216) compared to baseline (0 min). At 30 min and 60 min, CORT levels were increased compared to baseline, respectively, at 30 min (β = 17.192, SE = 3.943, *t* = 4.36, *p* < 0.001) and 60 min (β = 34.653, SE = 3.943, *t* = 8.788, *p* < 0.001).

Importantly, the interaction between time and mercury concentration was significant at 60 min (β = −20.009, SE = 4.181, *t* = -4.786, *p* < 0.001) and not at 15 min (β = −1.701, SE = 4.181, *t* = -0.407, *p* = 0.686) and 30 min (β = −7.598, SE = 4.181, *t* = −1.817, *p* = 0.075). These results show that the effect of mercury on CORT levels varies over time points, with a strong negative association at 60 min.

Mercury (THg) concentrations in the blood (RBCs) of *P. trigonatus* ranged from 0.317 to 1.982 µg.g^−1^ dw. Hg concentrations for the group Low ranged from 0.317 to 0.378 µg.g^−1^ dw; group Medium ranged from 0.586 to 0.899 µg.g^−1^ dw; and group High ranged from 1.001 to 1.982 µg.g^−1^ dw (Fig. 1B, Table S1). The repeated-measures ANOVA showed that the effect of group was not significant, F (2,17) = 2,90, *p* = 0.083, the effect of time was statistically significant, F (1.78, 30.33) = 46,31, *p* < 0.001, and the interaction between group and time was statistically significant, F (1.78, 30.33) = 7.26, *p* < 0.001, showing that the effect of time on CORT depends on the group.

We found significant differences (all *p* < 0.001, except group Low T15–T30 *p* = 0.018) in CORT levels between sampling time for the groups Medium and Low, apart from T0–T15 (*p* > 0.05). We did not find any significant differences in CORT levels between sampling time in the group High (Table S2).

We did not find a significant difference between the three groups for the CORT baseline “T0” ( < 3–5 min), the CORT levels at 15 min “T15”, 30 min “T30” (*p* > 0.05), however, we found a significant difference for the CORT levels at 60 min “T60” between the groups High and Low (*p* < 0.05), and High and Medium (*p* < 0.01) (Fig. 3 and Table S3). The amplitude of the stress response (∆CORT = T60–T0) was negatively related to blood Hg concentration (linear regression: R^2^ = 0.398, *p* = 0.003; Fig. 3).

## Discussion

The ability to respond to stressors is essential for adaptation and, ultimately, survival^22^. We evaluated the stress response of adult and subadult smooth-fronted caimans by measuring CORT release using a standardized stress protocol. The corticosteroid stress response is an endocrine response triggered by potentially threatening changes. It results from the activation of the HPA axis, which starts with the release of corticotropin-releasing hormone from the hypothalamus and ends with the secretion of glucocorticoids from the adrenals into the circulatory system. The ability to respond adequately to stress stimuli is determined by the amplitude of the secretion of adrenal steroids, such as CORT in birds and reptiles, and the sensitivity of neural circuits involved in the regulation of the HPA axis^23,24^. Various short-term stressors may induce physiological stress responses of different shapes, which in turn may lead to specific behavioral responses. However, chronic stressors such as environmental contaminants, can negatively impact the ability to mount an appropriate stress response. Chronic stress can also adversely affect reproductive physiology, impairing fertility and reproductive success^25,26^. In several species, chronic anthropogenic stress can modify the physiological stress response to new and unpredictable challenges^27,28^.

Physiological response to chronic stress is complex, and several studies have indicated that chronic stress can be partially counterbalanced by dampening down the sensitivity of the HPA axis^25,28^. Consequently, we expected that caimans with higher Hg blood levels, if this contaminant is inducing chronic stress, will reach lower concentrations of CORT in response to a stress event in comparison to non-chronically stressed individuals. The linear mixed-effects model shows a significant increase of CORT levels at 30 min (β = 17.192, SE = 3.943, t = 4.36, *p* < 0.001) and 60 min (β = 34.653, SE = 3.943, *t* = 8.788, *p* < 0.001) compared to baseline. Further, the decreasing interaction between Hg concentration and CORT levels is significant at 60 min(β = −20.009, SE = 4.181, *t* = -4.786, *p* < 0.001, Fig. 2).

For a complementary analysis, we allocated the individuals (*n* = 20) to three groups based on their blood Hg concentrations (Fig. 1B): Low (Hg ≤ 0.5 µg.g^−1^), Medium (0.5 < Hg < 1 µg.g^−1^), and High (Hg ≥ 1 µg.g^−1^).

A significant increase in CORT levels over time was observed in the Low and Medium groups (*p* < 0.05), except between T0 and T15 (*p* > 0.05). In contrast, CORT levels in the High group showed no significant change over time (Fig. 3A and Table S2). At baseline, 15- and 30-minute timepoints, CORT levels did not differ among groups (Fig. 3A and Table S3). However, at 60 min, CORT concentrations in the High group were significantly lower than those in the Low (p = 0.012) and Medium (*p* = 0.008) groups. Thus, both analytical approaches provide convergent results showing a down-regulation of the HPA axis associated with elevated Hg contamination.

The downregulation of the CORT response associated to elevated Hg concentrations indicates a long-term alteration of the HPA axis may be caused by Hg contamination. Such alteration can significantly impact individuals, as this system plays a critical role in regulating daily behaviors and reproductive activities^29,30^, and helps organisms to respond to acute environmental stressors, including extreme weather conditions, predation, and conspecific aggression^31^. Furthermore, disruptions of the HPA axis may have long-term consequences, potentially impairing the evolutionary capacity of species to adapt to habitat and ecological pressures^32^. By deteriorating the caimans’ ability to mount a proper stress response Hg contamination poses a threat to population survival on multiple levels.

We evaluated the relationship between the amplitude of the CORT response (the difference between baseline and peak CORT levels, ∆CORT) and blood Hg concentrations. ∆CORT informs on the ability of an individual to cope with a stressful event and maintain homeostasis, a measure of physiological flexibility to face unpredictable challenges. Here, the amplitude of the stress response had a negative relationship with concentrations of Hg found in the blood of caimans (R^2^ = 0.398, *p* = 0.003), suggesting a direct effect of Hg on the responsiveness of the HPA axis. The ∆CORT of the most contaminated individual was more than 100-fold lower than in the individual with the lowest Hg concentration (Fig. 3B). The inability to mount a physiological stress response likely prevents proper behavioral and physiological responses during threatening challenges.

**Fig. 4:**
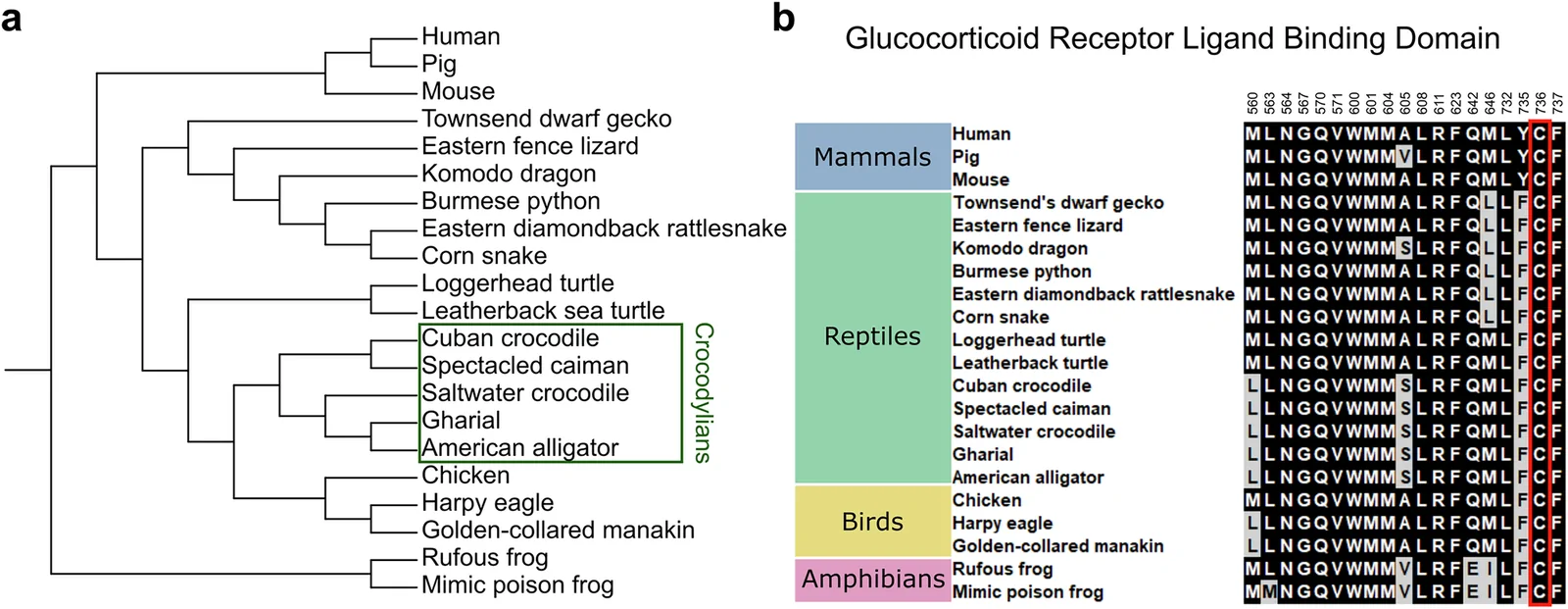
Phylogeny and conservation of the glucocorticoid receptor (GR) ligand-binding domain (LBD). **A** Phylogenetic tree based on ligand-binding domain of the glucocorticoid receptor. **B** Location of mercury-sensitive cysteine Cys736 (red box) shown among functional residues of the ligand-binding domain (LBD) of the glucocorticoid receptor. Aligned sequences show amino acids colored according to their comparison to sites in the human GR LBD sequence: with black background if they share an identical amino acid, or with a gray background if they have a different amino acid. Numbering corresponds to the position in the human GR (NP_000167.1). Accession numbers for all species are listed in Table S4, for the alignment of the complete LBD sequence see Fig. S2.

Heavy metals, such as cadmium, lead, and chromium, have been shown to interfere with steroid receptors^33,34^. Similarly, mercury could act as a glucocorticoid receptor (GR) antagonist. Glucocorticoid receptors and mineralocorticoid receptors are generally targets of EDCs, and recent work shows that metals interfere with them: Hg reduced GR hormone binding in male rats^35,36^ and altered the expression of GR-responsive genes^35–37^. The interaction between Hg and the GR protein causes inhibition of GR signaling, a major contribution to the neurotoxic effects of Hg^38^. In addition, in silico simulations show that mercuration of a cysteine residue (Cys736) located within the ligand-binding domain (LBD) of the human GR partially blocks access of the glucocorticoid analog dexamethasone to the ligand binding site^38^. This mechanism has been proposed to explain Hg’s ability to decrease GR activation by endogenous glucocorticoids, which could lead to lower expression of GR-driven genes^38^. As cysteine is a primary target of Hg, in its methylmercury form, due to its reactive thiol group, disrupting GR function through the mercuration of this particular cysteine could be responsible for the direct relationship we observed between Hg concentration and the amplitude of CORT in caimans. Notably, the presence of Cys736 is highly conserved across taxa in GR orthologs, including crocodylians (Fig. 4).

Currently, one-third of all crocodylian species are classified as threatened, making them one of the most endangered taxa on the planet. While various human activities drive this alarming decline, pollution through mining activities, stands out as a critical but underexplored global threat. Notably, crocodylian habitats often overlap with mining exploitations, exposing them to various toxic contaminants and habitat destruction^8^. Mercury is among the most concerning contaminants, as ASGM is one of the leading mining activities occurring in tropical and subtropical regions^39^. Despite the significant detrimental effects of Hg on human and wildlife endocrine systems^12^, its effects remain insufficiently explored in crocodylians. The observation of HPA axis alterations in this study at comparatively low Hg concentrations (between 0.317 and 1.982 µg.g^−1^ Hg) is notable, as these levels fall within the range reported in other crocodylian species worldwide^8^. For example, concentrations of up to 11 µg.g^−1^ have been detected in the blood of the Central African slender-snouted crocodile *Mecistops leptorhynchus* in Gabon, a critically endangered species in West Africa^40^. Furthermore, these low Hg concentration effects imply that even crocodylians living farther from ASGM sites may already be at risk of suffering mercury-induced cognitive and physiological impairments.

One of the few known examples of environmental contamination in crocodylians is the 1980 Lake Apopka (Florida, USA) chemical spill, which led to a decades-long dramatic decline in its population of American alligators *Alligator mississippiensis*. Failure to reproduce was attributed to organizational defects in the development of both male and female reproductive organs, illustrating the detrimental effects of chemical contamination on the endocrine system of crocodylians^41^. Endocrine disruption of the thyroid gland from Hg contamination has been shown in wild Western Pond Turtle *Emys marmorata*^42^. Previous work examining the direct link between Hg and CORT in reptiles is limited to two studies on crocodylians, both of which found that Hg concentrations negatively affected CORT levels^15,16^. Controversially, experimental studies on the effects of Hg on reptiles have failed to show severe sublethal consequences^43^. However, most of these studies have been conducted on juveniles with a short period of exposure to Hg, which does not reflect chronic exposure of animals in the wild. In fact, studies with experimental protocols that simulated prolonged environmental exposure demonstrated significant effects of low doses of EDCs on behavior and reproductive traits^44^.

In birds, Hg contamination has been shown to affect the endocrine and reproductive systems^45^; however, the link between chronic Hg exposure and endocrine disruption seems to be less straightforward than in the present study, as birds reduce their Hg body burden through molting. Nevertheless, the question of the long-term and global impacts of Hg as an endocrine disruptor remains unanswered in crocodylians. Here, our phylogenetic analysis of the GR suggests Hg may have the ability to affect the HPA axis via a shared cysteine-mediated mechanism, regardless of species. Unlike most vertebrates, which face environmental change over several generations, crocodylians may face such changes within the span of a single generation. In most large terrestrial vertebrates, generation length typically ranges from 5 to 10 years, corresponding to approximately 20 generations since the pre-industrial era (1850–1900). In contrast, this entails only 5 generations in crocodylians. The extended generation time of long-lived taxa can make it challenging to detect detrimental effects and may partly explain the difficulty in assessing population trends in crocodylians. Given the key role of the endocrine system in mediating physiological adaptations and reproductive success, the present results highlight the sensitivity of the crocodylian HPA axis to Hg contamination, stress the harmful effects of ASGM activities on the taxon, and ultimately emphasize the value of research on long-lived taxa for understanding chronic effects of environmental contaminants.

## Methods

### Sample collection

The study was conducted in French Guiana between November 2023 and June 2024. A diversity of sampling sites was included, ranging from pristine rainforest to ASGM sites including Mont-Grant Matoury nature reserve, Nouragues nature reserve (Parare, Inselberg, Orion) and Petit-Saut, providing a gradient of mercury (Hg) contamination. We captured and sampled 20 adult and sub-adult smooth-fronted caimans, *Paleosuchus trigonatus*, between 08:00 pm and 11:00 pm; the 3-h window was set to reduce the impact of the circadian rhythm on sampling. We took care not to attempt any captures near hatchlings or apparent nesting sites. Captures were performed using a snare pole. After capture, the snout of the individual was closed and secured using electrical tape, and a wet towel was placed on the head to cover the eyes and standardize the stress event. Serial blood sampling was performed on all individuals at < 3–5 min (T0), 15 min (T15), 30 min (T30), and 60 min (T60) after capture to assess the stress response. Blood was taken from the occipital cranial vein using a sterile 3 mL syringe and a 25-gauge heparinized needle (heparin sodium). Each blood sample (0.3 to 1 mL) was centrifuged at 6500 rpm for 5 min to separate red blood cells (RBCs) for Hg, and plasma for corticosterone (CORT) quantification. Using a micropipette, 20–100 µL of plasma was transferred to a tube containing 750 µL of 99.8% ethanol (HPLC grade). RBC samples were stored in 96% ethanol until analysis in the lab. Capture of animals, sample collection and transportation were performed under permit from the French authorities (Direction Régionale des Territoires et de la Mer) after evaluation by the CSRPN, the regional scientific committee (Permits: R03-2022-10-20-00004).

### Mercury quantification

Before analysis, ethanol from RBC samples was evaporated under an N_2_ stream at room temperature; the samples were freeze-dried for 48 h, and then ground and homogenized. Total Hg (THg) was quantified by direct measurement of at least two replicates using a Direct Mercury Analyser (DMA-80 evo III, atomic absorption spectrometer, Milestone) at the University of Vienna, Austria. Inter-replicate reproducibility for each sample was validated against a relative standard deviation threshold of <10%. The method was validated by analysis of certified reference material (CRM) of TORT-3 (Lobster hepatopancreas, National Research Council of Canada; certified reference Hg concentration: 0.292 ± 0.022 µg.g^−1^ dry weight (dw)). CRM was analyzed at the beginning and end of the analytical cycle, as well as after every ten samples. Measured values of TORT-3 were 0.298 ± 0.001 µg.g^−1^ dw (*n* = 5), with a recovery rate of 102.0 ± 0.5%. The detection limit of the DMA-80 was 0.01 ng. Hg concentrations are expressed in µg.g^−1^ dw.

### Corticosterone quantification

Samples were brought to room temperature, briefly vortexed, and centrifuged at 3900 rpm for 10 min to separate the solids from the liquid phase. The supernatant was carefully removed and pipetted into a glass tube. Next, 750 µL of absolute ethanol (96%) was added to the sample tube, briefly vortexed, and centrifuged again at 3900 rpm for 10 min to separate the solids from the liquid phase. The supernatant was combined with the first liquid phase from the same sample and dried under an N_2_ stream at 37 °C.

For the liquid-liquid extraction (LLE), the samples were resuspended in 800 µL of ultrapure water, and 4 mL of dichloromethane (HPLC grade) was added. The glass tubes were capped, and the samples were shaken for 4 h at room temperature. Then, samples were centrifuged for 10 min at 3900 rpm to separate the organic from the water phases. The organic phase was decanted into a new glass tube after LLE using a dry ice bath. Another 4 mL of dichloromethane was added to the thawed water phase, vortexed, separated by freezing in a dry ice bath, and combined with the dichloromethane phase from the first LLE. The dichloromethane phase was dried entirely under an N_2_ stream at 37°C.

The dry samples were resuspended in 150–400 µL of assay buffer provided by the ELISA manufacturer (Arbor Assays assay buffer X065). The volumes used depended on the dilution factors, which ranged from 1:10 to 1:25, depending on the initial amount of plasma. The samples were stored overnight at 4 °C until being assayed the next day.

Samples were measured with Arbor Assays DetectX® Corticosterone Multi-Format ELISA Kits (Cat. No. K014-H). The assay was performed according to the manufacturer’s instructions, using the 100 µL protocol, which resulted in a higher assay sensitivity than the 50 µL protocol (standard curve range 19–5000 pg/mL vs. 39–10000 pg/mL, respectively).

Briefly, 100 µL of each sample extract and 25 µL of each conjugate and antibody were added to the plate. After incubation, the plates were washed, and 100 µL of tetramethylbenzidine substrate solution was added. After the second incubation, plates were stopped with the provided stop solution, and photometric absorption was measured at 450 nm using a spectrophotometer (Thermo Fisher Multiskan Go 51119200/Type 1510).

All samples and standards were measured in duplicate, and the coefficient of variation between duplicate measurements was below 5% for all. The mean concentration of duplicate measurements corrected for the dilution factor was used in subsequent calculations for samples. All samples from one individual were measured on the same plate. All samples were measured between 10 and 80% of maximum binding, assuring accurate determination of corticosterone levels. The detection limit for each plate was calculated at two standard deviations from the B0 along the standard curve. No samples were measured below the detection limits of the respective plate (mean % binding 96%, ~15 pg/mL corticosterone).

Two different control samples were run on each plate of the two sample plates to assess the variation between plates and extractions. The quality control sample consisted of Arbor Assays assay buffer X065, to which corticosterone was added for a final concentration of 400 pg/mL. This sample was run twice in duplicate on each plate. Inter-assay variation across plates was 12%, and intra-assay variation within plates was 4% for this sample. To estimate extraction efficiency and variation between extraction batches and plates, we spiked stripped chicken plasma with corticosterone standard for a concentration of 20,000 pg/mL. We then used 10 µL of this extraction control sample, added 200 µL ultrapure water for volume, and extracted this sample with every extraction batch alongside the samples, using the same procedure as above. The dried sample was reconstituted with 390 µL assay buffer to a final corticosterone concentration of ~500 pg/mL. This sample was run in duplicate with each extraction batch/plate. Inter-assay variation for the extraction control sample was 4%, and mean extraction efficiency (% of observed vs. expected concentration) was 105%, indicating that the extraction procedure was consistent and yielded accurate measurements. The quantification of CORT was performed at the University of Vienna, Austria.

### Statistical analysis

All statistical analyses were performed using the software R v.4.4.0 (R Core Team). All data were checked for normality and homogeneity of variance.

To evaluate the effect of Hg concentration on the stress response, we used two different approaches. First, we used a linear mixed-effects model with CORT level as the dependent variable, and the fixed effects included time (as a categorical variable with levels T0, T15, T30 and T60), Hg concentration, and their interaction (time × Hg). The interaction term was included to assess whether the effect of Hg on CORT levels varied across time points. A random intercept for each individual was included to account for repeated measures within individuals. The model was fit using restricted maximum likelihood estimation with the ‘lme4’ package. Statistical significance of fixed effects was assessed using the Satterthwaite approximation for degrees of freedom, as implemented in the ‘lmerTest’ package. Model assumptions were checked by visually inspecting residual plots for normality and homoscedasticity. We then fitted a second linear mixed-effects model including sex, total length, and sampling date as additional covariates, which were not significant.

Second, using blood Hg concentrations results from all individuals, three groups were formed: first, individuals under 0.5 µg.g^−1^, which can be considered as environmental background levels^46^ called Low, second, individuals between 0.5 and 1 µg.g^−1^ called Medium, and third, individuals over 1 µg.g^-1^ called High. We used repeated measures analysis of variance (ANOVA) to assess differences across groups and time points. Post-hoc pairwise comparisons were performed using Bonferroni correction to adjust for multiple comparisons. The relationship between the amplitude of CORT and log-transformed Hg concentrations was calculated using linear regression.

### Phylogenetic analysis of the ligand-binding domain of the glucocorticoid receptor

Mercuration of Cys736 in the human glucocorticoid receptor (GR) ligand-binding domain (LBD) has been shown to interfere with binding of the glucocorticoid analog dexamethasone in in silico simulations and in vitro assays^38^. To assess whether Cys736 in the human GR-LBD was conserved in caimans and other species, we compiled GR protein sequences from 21 species using two methods (Table S4). First, we used the human GR protein sequence (NP_000167.1) as query in the Protein BLAST server and selected representative species for mammals, birds, amphibians, and reptiles. In addition, because we sought to collect as many crocodylian sequences as possible, we selected all non-redundant sequences after applying the filter “crocodiles and alligators”. Second, we used the GR-LBD as query in the tBLASTn server (residue positions 531-777; ‘NR_GR_LBN’ cd07076, conserved domain database CDD^47^) against the spectacled caiman and Cuban crocodile genomes, UST_Ccro_1.0 and ASM3850303v1, respectively. We then manually concatenated the top hits for these two species.

Sequences were aligned using the Constraint-based Multiple Alignment Tool (NCBI COBALT server). Because we had only recovered the LBD portion of the GR sequence for the Burmese python, spectacled caiman, and Cuban crocodile, we used this region to calculate the phylogenetic tree within COBALT. The Newick tree file from COBALT was modified for aesthetics in the Interactive Tree of Life server (iTOL v.7.2.2)^48^.

To assess conserved functional residues for ligand binding, we submitted all sequences to a batch CD-search with default settings^49,50^, confirming that 18 out of the 18 functional residues in the NR_GR_LBD domain were recognized as conserved in the majority of species; full results are shown in data S1. We show a summary of these results complemented with information from the aligned sequences in Fig. 4B. To prepare the colored alignment showing the entire LBD for the entire dataset (Fig. S2) we used the color align conservation tool at bioinformatics.org^51^ with the threshold for coloring identity and similarity set to 90% and re-numbered the starting positions to ensure each sequence was annotated with species-specific position numbers corresponding to their respective GR protein sequence.

### Reporting summary

Further information on research design is available in the Nature Portfolio Reporting Summary linked to this article.

## Supplementary information

Supplementary Material

Description of Additional Supplementary Files

Supplementary Data

Reporting Summary

## Data Availability

All data supporting the finding of this study are available within the paper and its Supplementary Information files. The source data underlying the figures can be found in Supplementary Data.
