# Supplementary material for: Mercury contamination impairs the hypothalamic-pituitary-adrenal axis of a neotropical caiman
Source: Commun Biol. 2026 Oct 28;9:1385. doi: 10.1038/s42003-026-11022-0 (PMC13716903; doi:10.1038/s42003-026-11022-0)
Supplement: Supplementary file 1 [file 42003_2026_11022_MOESM1_ESM.pdf]

Supplementary Information for

Mercury contamination impairs the hypothalamic-pituitary-adrenal axis of a neotropical caiman

Jérémy Lemaire, Rosanna Mangione, Jasmine L. Loveland, Leonida Fusani, Virginie Canoine

Corresponding author: jeremy.lemaire@univie.ac.at

The PDF file includes:

Figs. S1 to S2
Tables S1 to S4
References (*47*-*52*)

Other Supplementary Materials for this manuscript include the following:

Data S1

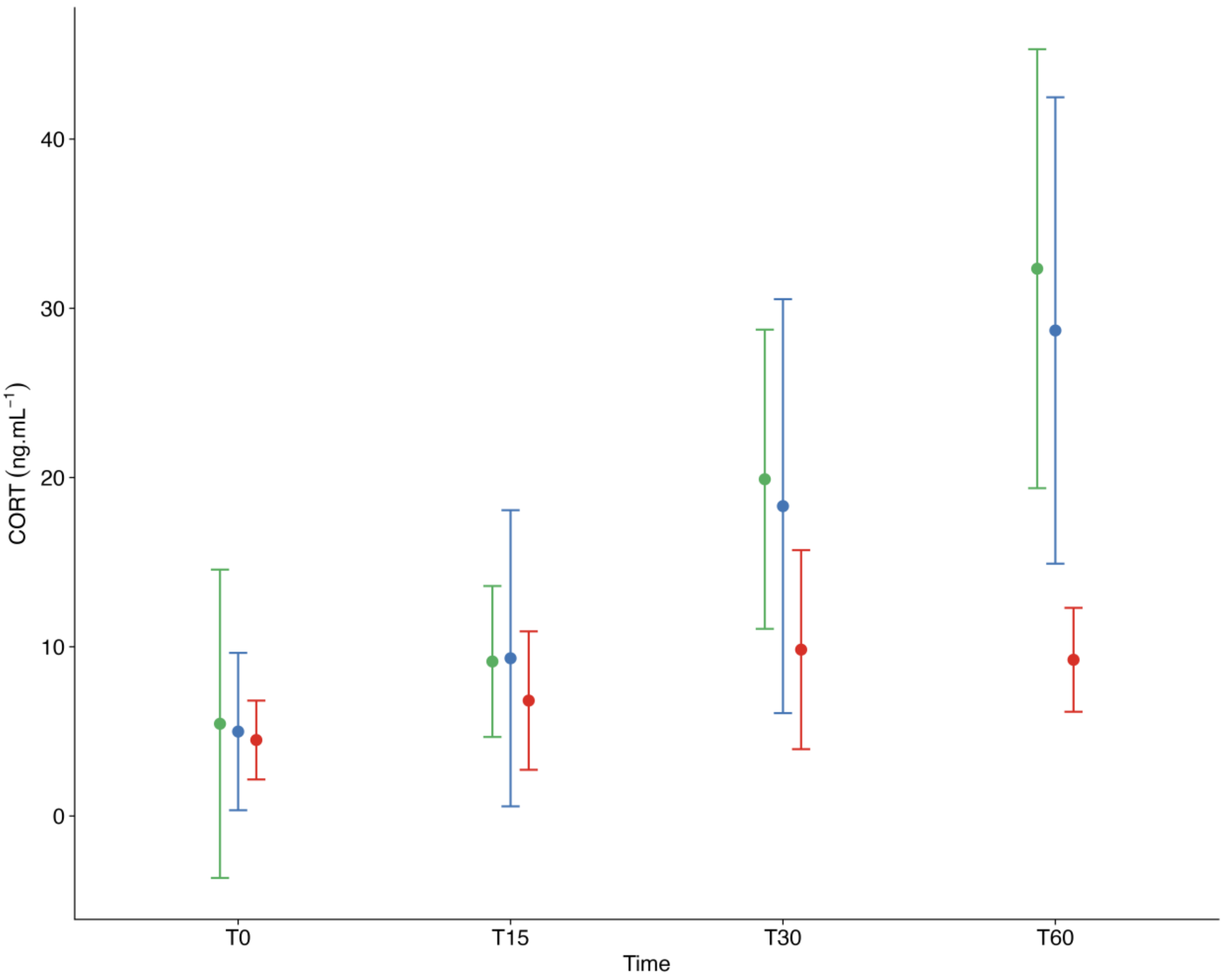

Fig. S1. Corticosterone stress response profile by sampled time points.
Mean ± SD corticosterone (CORT) levels in groups High (Hg > 1 µg.g^{-1}, red), Medium (0.5 < Hg < 1 µg.g^{-1}, blue) and Low (Hg < 0.5 µg.g^{-1}, green) at each time sampling point. Significant difference for the CORT levels at 60 mins "T60" were found between the groups High and Low ($p > 0.05$) and High and Medium ($p < 0.01$). Abbreviations: T0, < 3-5 minutes; T15, 15 minutes; T30, 30 minutes; T60, 60 minutes; Hg, Mercury.

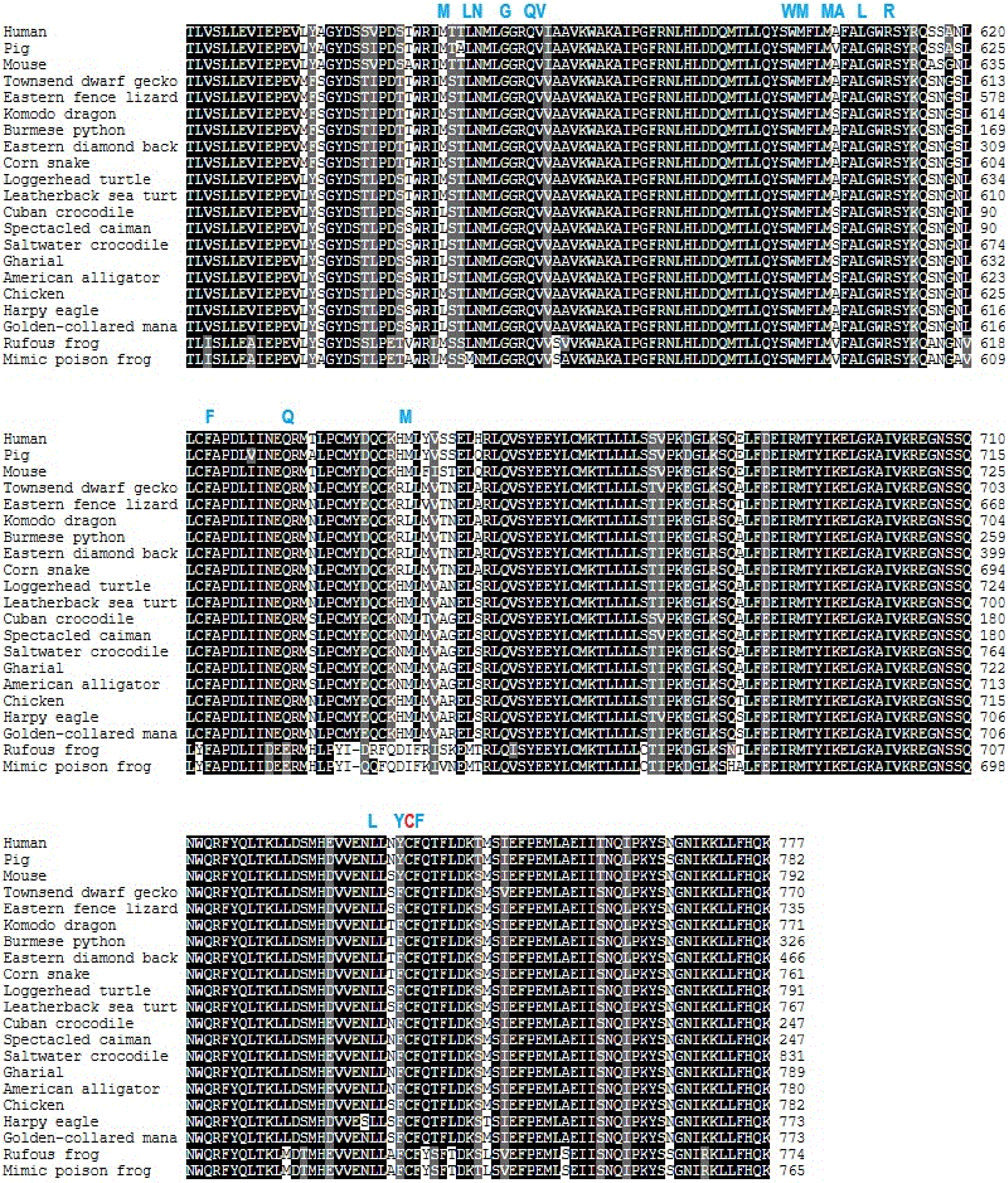

Fig. S2. Alignment of amino acid sequences from the ligand binding domain (LBD) of the glucocorticoid receptor (GR). Aligned sequences show the ligand-binding domain of the glucocorticoid receptor is highly conserved across species spanning across major taxa in vertebrates, including crocodylians. The 18 functional residues that form part of the canonical NR_GR_LBD (Conserved Domains Database ID, cd07076) are shown in blue letters at their respective positions in the alignment. The cysteine at position 736 in the human LBD, which has been shown to disrupt ligand binding when mercurated (*39*), is shown in red.

	N	T0 (ng.mL^{-1})	T15 (ng.mL^{-1})	T30 (ng.mL^{-1})	T60 (ng.mL^{-1})	Hg (µg.g^{-1})
**Low**						
Mean ± SD	4	5.45 ± 9.11	9.13 ± 4.46	19.90 ± 8.84	32.34 ± 12.97	0.350 ± 0.027
[Min-Max]		0.48 – 19.1	6.65 – 15.8	11.7 – 31.5	17.24 – 44.75	0.317 – 0.378
**Medium**						
Mean ± SD	9	4.99 ± 4.65	9.33 ± 8.75	18.31 ± 12.23	28.69 ± 13.78	0.702 ± 0.090
[Min-Max]		1.00 – 12.63	1.40 – 25.87	3.91 – 33.39	8.20 – 46.51	0.586 – 0.899
**High**						
Mean ± SD	7	4.50 ± 2.33	6.82 ± 4.09	9.83 ± 5.88	9.23 ± 3.07	1.318 ± 0.325
[Min-Max]		1.81 – 8.82	2.47 – 14.36	2.97 – 20.21	5.72 – 13.90	1.000 – 1.982

Table S1: Corticosterone levels at time sampling points in each mercury concentration group.

Abbreviations: T0, < 3-5 minutes from capture; T15, 15 minutes; T30, 30 minutes; T60, 60 minutes; Hg, Mercury.

**Low**				
	T0	T15	T30	T60
T0	-	$t(17) = -1.500$, $p = 0.912$	$t(17) = -3.970$, $p = 0.006$	$t(17) = -28.892$, $p < 0.001$
T15	$t(17) = -1.500$, $p = 0.912$	-	$t(17) = -10.762$, $p = 0.018$	$t(17) = -23.212$, $p < 0.001$
T30	$t(17) = -3.970$, $p = 0.006$	$t(17) = -10.762$, $p = 0.018$	-	$t(17) = -12.450$, $p = 0.002$
**Medium**				
	T0	T15	T30	T60
T0	-	$t(17) = -4.336$, $p = 0.101$	$t(17) = -13.324$, $p < 0.001$	$t(17) = -23.699$, $p < 0.001$
T15	$t(17) = -4.336$, $p = 0.101$	-	$t(17) = -8.989$, $p = 0.003$	$t(17) = -19.363$, < 0.001
T30	$t(17) = -13.324$, $p < 0.001$	$t(17) = -8.989$, $p = 0.003$	-	$t(17) = -10.374$, $p < 0.001$
**High**				
	T0	T15	T30	T60
T0	-	$t(17) = -2.316$, $p = 1.000$	$t(17) = -5.340$, $p = 0.414$	$t(17) = -4.731$, $p = 1.000$
T15	$t(17) = -2.316$, $p = 1.000$	-	$t(17) = -3.024$, $p = 1.000$	$t(17) = -2.416$, $p = 1.000$
T30	$t(17) = -5.340$, $p = 0.414$	$t(17) = -3.024$, $p = 1.000$	-	$t(17) = 0.609$, $p = 1.000$

Table S2: Post-hoc comparisons of corticosterone concentrations between time points within groups.

Differences in corticosterone levels between sampling time points for each group were examined using repeated-measures ANOVA followed by post-hoc pairwise comparisons. Reported values are Bonferroni-corrected p-values. Abbreviations: T0, < 3-5 minutes from capture; T15, 15 minutes; T30, 30 minutes; T60, 60 minutes.

**T0**			
	Low	Medium	High
Low	-	t(17) = 0.463, p= 1.000	t(17) = 0.951, p = 1.000
Medium	t(17) = 0.463, p= 1.000	-	t(17) = 0.489, p = 1.000
**T15**			
	Low	Medium	High
Low	-	t(17) = -0.193, p = 1.000	t(17) = 2.315, p = 1.000
Medium	t(17) = -0.193, p = 1.000	-	t(17) = 8.473, p = 1.000
**T30**			
	Low	Medium	High
Low	-	t(17) = 1.581, p = 1.000	t(17) = 10.054, p = 0.362
Medium	t(17) = 1.581, p = 1.000	-	t(17) = 8.473, p = 0.315
**T60**			
	Low	Medium	High
Low	-	t(17) = 3.656, p = 1.000	t(17) = 23.112, p = 0.012
Medium	t(17) = 3.656, p = 1.000	-	t(17) = 19.456, p = 0.008

Table S3: Post-hoc comparisons of corticosterone concentrations between groups at each time point.

A significant difference in corticosterone levels between sampling time points for each group was examined using repeated-measures ANOVA followed by post-hoc pairwise comparisons. Reported values are Bonferroni-corrected p-values. Abbreviations: T0, < 3-5 minutes from capture; T15, 15 minutes; T30, 30 minutes; T60, 60 minutes.

Accession #	Protein name	Species	Common name	Length
KAK9406600.1	Glucocorticoid receptor	*Crotalus adamanteus*	Eastern diamondback rattlesnake	466
None	Glucocorticoid receptor, partial	*Caiman crocodilus*	Spectacled caiman	247
None	Glucocorticoid receptor, partial	*Crocodylus rhombifer*	Cuban crocodile	247
NP_000167.1	Glucocorticoid receptor	*Homo sapiens*	Human	777
NP_001008481.1	Glucocorticoid receptor	*Sus scrofa*	Pig	782
NP_001032915.1	Glucocorticoid receptor	*Gallus gallus*	Chicken	772
NP_001274234.1	Glucocorticoid receptor	*Alligator mississippiensis*	American alligator	780
NP_001348138.1	Glucocorticoid receptor	*Mus musculus* strain C57BL/6	Mouse	792
XP_007435648.1	Glucocorticoid receptor, partial	*Python bivittatus*	Burmese python	326
XP_008926174.1	Glucocorticoid receptor	*Manacus vitellinus*	Golden-collared manakin	773
XP_019361908.1	PREDICTED: glucocorticoid receptor isoform X1	*Gavialis gangeticus*	Gharial	789
XP_019390549.1	PREDICTED: glucocorticoid receptor	*Crocodylus porosus*	Saltwater crocodile	831
XP_034260821.1	Glucocorticoid receptor	*Pantherophis guttatus*	Corn snake	761
XP_038267932.1	Glucocorticoid receptor	*Dermochelys coriacea*	Leatherback sea turtle	767
XP_042303449.1	Glucocorticoid receptor	*Sceloporus undulatus*	Eastern fence lizard	735
XP_044310498.1	Glucocorticoid receptor isoform X2	*Varanus komodoensis*	Komodo dragon	771
XP_048347372.1	Glucocorticoid receptor	*Sphaerodactylus townsendi*	Townsend’s dwarf gecko	770
XP_048715885.1	Glucocorticoid receptor isoform X1	*Caretta caretta*	Loggerhead turtle	791
XP_052672243.1	Glucocorticoid receptor	*Harpia harpyja*	Harpy eagle	773
XP_069620158.1	Glucocorticoid receptor	*Ranitomeya imitator*	Mimic poison frog	765
XP_075131113.1	Glucocorticoid receptor isoform X2	*Leptodactylus fuscus*	Rufous frog	774

Table S4: Glucocorticoid receptor protein sequences used in this study.
List of accession numbers for glucocorticoid receptor protein sequences used to assemble a phylogenetic tree and examine conserved residues within the ligand-binding domain. For the assembly of spectacled caiman and Cuban crocodile sequences see Supplementary Methods.

Data S1. (separate file)
