# Supplementary material for: Mercury contamination impairs the hypothalamic-pituitary-adrenal axis of a neotropical caiman
Source: Commun Biol. 2026 Oct 28;9:1385. doi: 10.1038/s42003-026-11022-0 (PMC13716903; doi:10.1038/s42003-026-11022-0)
Supplement: Supplementary file 2 [file 42003_2026_11022_MOESM2_ESM.pdf]

Description of Additional Supplementary Files:

File: Supplementary Data

Description: The source data underlying the figures
