# Supplementary material for: Mercury contamination impairs the hypothalamic-pituitary-adrenal axis of a neotropical caiman
Source: Commun Biol. 2026 Oct 28;9:1385. doi: 10.1038/s42003-026-11022-0 (PMC13716903; doi:10.1038/s42003-026-11022-0)
Supplement: Supplementary file 3 [file 42003_2026_11022_MOESM4_ESM.pdf]

nature portfolio

Corresponding author(s): Jérémy Lemaire

Last updated by author(s): Jul 15, 2026

Reporting Summary

Nature Portfolio wishes to improve the reproducibility of the work that we publish. This form provides structure for consistency and transparency in reporting. For further information on Nature Portfolio policies, see our Editorial Policies and the Editorial Policy Checklist.

Statistics

For all statistical analyses, confirm that the following items are present in the figure legend, table legend, main text, or Methods section.

n/a	Confirmed	
☐	☒	The exact sample size (*n*) for each experimental group/condition, given as a discrete number and unit of measurement
☐	☒	A statement on whether measurements were taken from distinct samples or whether the same sample was measured repeatedly
☐	☒	The statistical test(s) used AND whether they are one- or two-sided<br>*Only common tests should be described solely by name; describe more complex techniques in the Methods section.*
☐	☒	A description of all covariates tested
☐	☒	A description of any assumptions or corrections, such as tests of normality and adjustment for multiple comparisons
☐	☒	A full description of the statistical parameters including central tendency (e.g. means) or other basic estimates (e.g. regression coefficient) AND variation (e.g. standard deviation) or associated estimates of uncertainty (e.g. confidence intervals)
☐	☒	For null hypothesis testing, the test statistic (e.g. *F*, *t*, *r*) with confidence intervals, effect sizes, degrees of freedom and *P* value noted<br>*Give P values as exact values whenever suitable.*
☒	☐	For Bayesian analysis, information on the choice of priors and Markov chain Monte Carlo settings
☒	☐	For hierarchical and complex designs, identification of the appropriate level for tests and full reporting of outcomes
☒	☐	Estimates of effect sizes (e.g. Cohen's *d*, Pearson's *r*), indicating how they were calculated

Our web collection on statistics for biologists contains articles on many of the points above.

Software and code

Policy information about availability of computer code

Data collection	no software was used
Data analysis	Software R v.4.4.0

For manuscripts utilizing custom algorithms or software that are central to the research but not yet described in published literature, software must be made available to editors and reviewers. We strongly encourage code deposition in a community repository (e.g. GitHub). See the Nature Portfolio guidelines for submitting code & software for further information.

Data

Policy information about availability of data

All manuscripts must include a data availability statement. This statement should provide the following information, where applicable:

- Accession codes, unique identifiers, or web links for publicly available datasets
- A description of any restrictions on data availability
- For clinical datasets or third party data, please ensure that the statement adheres to our policy

All code and materials used in the analysis are available at https://phaidra.univie.ac.at/o:2203202

Research involving human participants, their data, or biological material

Policy information about studies with human participants or human data. See also policy information about sex, gender (identity/presentation), and sexual orientation and race, ethnicity and racism.

Reporting on sex and gender	*Use the terms sex (biological attribute) and gender (shaped by social and cultural circumstances) carefully in order to avoid confusing both terms. Indicate if findings apply to only one sex or gender; describe whether sex and gender were considered in study design; whether sex and/or gender was determined based on self-reporting or assigned and methods used. Provide in the source data disaggregated sex and gender data, where this information has been collected, and if consent has been obtained for sharing of individual-level data; provide overall numbers in this Reporting Summary. Please state if this information has not been collected. Report sex- and gender-based analyses where performed, justify reasons for lack of sex- and gender-based analysis.*
Reporting on race, ethnicity, or other socially relevant groupings	*Please specify the socially constructed or socially relevant categorization variable(s) used in your manuscript and explain why they were used. Please note that such variables should not be used as proxies for other socially constructed/relevant variables (for example, race or ethnicity should not be used as a proxy for socioeconomic status). Provide clear definitions of the relevant terms used, how they were provided (by the participants/respondents, the researchers, or third parties), and the method(s) used to classify people into the different categories (e.g. self-report, census or administrative data, social media data, etc.) Please provide details about how you controlled for confounding variables in your analyses.*
Population characteristics	*Describe the covariate-relevant population characteristics of the human research participants (e.g. age, genotypic information, past and current diagnosis and treatment categories). If you filled out the behavioural & social sciences study design questions and have nothing to add here, write "See above."*
Recruitment	*Describe how participants were recruited. Outline any potential self-selection bias or other biases that may be present and how these are likely to impact results.*
Ethics oversight	*Identify the organization(s) that approved the study protocol.*

Note that full information on the approval of the study protocol must also be provided in the manuscript.

Field-specific reporting

Please select the one below that is the best fit for your research. If you are not sure, read the appropriate sections before making your selection.

☐ Life sciences ☐ Behavioural & social sciences ☒ Ecological, evolutionary & environmental sciences

For a reference copy of the document with all sections, see nature.com/documents/nr-reporting-summary-flat.pdf

Ecological, evolutionary & environmental sciences study design

All studies must disclose on these points even when the disclosure is negative.

Study description	We evaluated the effect of mercury concentrations on the capacity of wild population of Smooth-fronted caiman, Paleosuchus trigonatus, to regulate corticosterone release following a stressful event.
Research sample	Wild adults and subadults of the smooth-fronted caiman, Paleosuchus trigonatus, were caught in French Guiana at different sampling sites, ranging from pristine rainforest to artisanal small-scale goldmining sites, providing a gradient of mercury contamination.
Sampling strategy	No sample size was calculated because all encountered individuals were captured, given the low population density and the difficulty of detection.
Data collection	Data collection was performed by Jérémy Lemaire and Rosanna Mangione.
Timing and spatial scale	The study was conducted between November 2023 and June 2024. Captures were performed between 20:00 and 23:00; this 3-hour window was chosen to minimize the influence of circadian rhythms on sampling.
Data exclusions	No data were excluded from the analysis.
Reproducibility	After capture, the snout of the individual was closed and secured using electrical tape, and a wet towel was placed on the head to cover the eyes and standardize the stress event.
Randomization	All encountered individuals were captured.
Blinding	Blinding was not relevant to the study.

Did the study involve field work? ☒ Yes ☐ No

Field work, collection and transport

Field conditions	All captures were performed between the end of the dry season and the end of the rainy season, meaning that samples were collected during most of the rainy season in French Guiana.
Location	Sampling was performed in French Guiana.
Access & import/export	Transport of sample were performed under permit from the French authorities (Direction Régionale des Territoires et de la Mer) after evaluation by the CSRPN, the regional scientific committee (Permits: R03-2022-10-20-00004).
Disturbance	Performing a stress-response study causes disturbances that are inevitable.

Reporting for specific materials, systems and methods

We require information from authors about some types of materials, experimental systems and methods used in many studies. Here, indicate whether each material, system or method listed is relevant to your study. If you are not sure if a list item applies to your research, read the appropriate section before selecting a response.

Materials & experimental systems

n/a	Involved in the study
☒	☐ Antibodies
☒	☐ Eukaryotic cell lines
☒	☐ Palaeontology and archaeology
☐	☒ Animals and other organisms
☒	☐ Clinical data
☒	☐ Dual use research of concern
☒	☐ Plants

Methods

n/a	Involved in the study
☒	☐ ChIP-seq
☒	☐ Flow cytometry
☒	☐ MRI-based neuroimaging

Animals and other research organisms

Policy information about studies involving animals; ARRIVE guidelines recommended for reporting animal research, and Sex and Gender in Research

Laboratory animals	The study did not evolve laboratory animals.
Wild animals	We captured wild adult and subadult smooth-fronted caimans (Paleosuchus trigonatus). Captures were performed using a snare pole. All individuals were released after sampling.
Reporting on sex	Findings apply to both sex.
Field-collected samples	Serial blood sampling was performed on all individuals at < 3-5 minutes, 15 minutes, 30 minutes, and 60 minutes after capture to assess the stress response. Blood was taken from the occipital cranial vein using a sterile 3 ml syringe and a 25-gauge heparinized needle (heparin sodium). Each blood sample (0.3 to 1 ml) was centrifuged at 6500 rpm for 5 minutes to separate red blood cells (RBCs) for Hg, and plasma for corticosterone (CORT) quantification. Using a micropipette, 20 - 100 µL of plasma was transferred to a tube containing 750 µL of 99.8% ethanol (HPLC grade). RBC samples were stored in 96% ethanol until analysis in the lab.
Ethics oversight	Capture of animals and sample collection were performed under permit from the French authorities (Direction Régionale des Territoires et de la Mer) after evaluation by the CSRPN, the regional scientific committee (Permits: R03-2022-10-20-00004).

Note that full information on the approval of the study protocol must also be provided in the manuscript.

Plants

Seed stocks

Report on the source of all seed stocks or other plant material used. If applicable, state the seed stock centre and catalogue number. If plant specimens were collected from the field, describe the collection location, date and sampling procedures.

Novel plant genotypes

Describe the methods by which all novel plant genotypes were produced. This includes those generated by transgenic approaches, gene editing, chemical/radiation-based mutagenesis and hybridization. For transgenic lines, describe the transformation method, the number of independent lines analyzed and the generation upon which experiments were performed. For gene-edited lines, describe the editor used, the endogenous sequence targeted for editing, the targeting guide RNA sequence (if applicable) and how the editor was applied.

Authentication

Describe any authentication procedures for each seed stock used or novel genotype generated. Describe any experiments used to assess the effect of a mutation and, where applicable, how potential secondary effects (e.g. second site T-DNA insertions, mosiacism, off-target gene editing) were examined.
